# Efficacy of a Novel Bioactive Glass-Polymer Composite for Enamel Remineralization following Erosive Challenge

**DOI:** 10.1155/2022/6539671

**Published:** 2022-04-22

**Authors:** Farnoosh Fallahzadeh, Soolmaz Heidari, Farhood Najafi, Maryam Hajihasani, Nooshin Noshiri, Neda F. Nazari

**Affiliations:** ^1^Department of Operative Dentistry, Faculty of Dentistry, Qazvin University of Medical Sciences, Qazvin, Iran; ^2^Department of Operative Dentistry, Dental Caries Prevention Research Center, Qazvin University of Medical Sciences, Qazvin, Iran; ^3^Department of Resin and Additives, Institute for Color Science and Technology, P.O. Box: 16765-654, Tehran, Iran; ^4^Postgraduate Student of Operative Dentistry, Student Research Committee, Qazvin University of Medical Sciences, Qazvin, Iran; ^5^Medical Image Processing Laboratory, Alzahra University, Tehran, Iran; ^6^Sharif University of Technology, Central Lab, Tehran, Iran

## Abstract

**Introduction:**

Dental caries is the most common cause of tooth loss. However, it can be stopped by enhancing remineralization. Fluoride and casein phosphopeptide-amorphous calcium phosphate (CPP-ACP) are among the most important remineralizing agents. Recent studies have used bioactive glass as a remineralizing agent in different forms. This study aimed to assess the efficacy of a composite paste (prepared by mixing urethane polypropylene glycol oligomer with bioactive glass powder for easier application).

**Materials and Methods:**

Enamel disks were cut out of the buccal surface of extracted sound third molars. The samples were randomly divided into 3 groups of 15 and underwent Vickers microhardness test. X-ray diffraction (XRD) and field emission scanning electron microscopy/energy dispersive X-ray spectroscopy (FESEM/EDS) were performed. All samples were immersed in a demineralizing solution for 14 days. The tests were then repeated. Next, bioactive glass paste, fluoride, and CPP-ACP were applied on the surface of the samples and they were then stored in an artificial saliva for 14 days. The tests were repeated again. The microhardness values were analyzed using repeated measures ANOVA followed by one-way ANOVA and Tukey's post hoc test (*P* < 0.05).

**Results:**

The microhardness of the bioactive glass group was significantly higher than that of other groups (*P* < 0.05). XRD revealed an enamel structure more similar to sound enamel in the bioactive glass and CPP-ACP groups compared with the fluoride group. FESEM/EDS revealed higher hydroxyapatite deposition in the bioactive glass group than in the other two groups.

**Conclusions:**

All three remineralizing agents caused remineralization, but bioactive glass paste had a greater efficacy.

## 1. Introduction

In the past, dental caries was thought to be an irreversible bacterial disease, but at present, it is considered a dynamic and reversible process. The role of remineralization should be evaluated and emphasized to control dental caries [1].

The human saliva contains calcium and phosphate ions in supersaturated state. Thus, it has the potential to remineralize enamel [2].

Poor success of cariostatic agents is due to their limited remineralizing potential in the presence of low concentrations of calcium and phosphate ions in the saliva since there is no source for such ions in the oral cavity [3]. Thus, researchers are searching for new remineralizing agents to serve as a rich source of ions. Bioactive glass and casein phosphopeptide-amorphous calcium phosphate (CPP-ACP) are among the currently available remineralizing agents. CPP-ACP is a bioactive material, which is composed of casein phosphopeptide (a milk protein) and amorphous calcium phosphate, and increases the salivary concentration of calcium and phosphate ions and their penetration into demineralized enamel crystals, causing remineralization of enamel lesions [4].

Following the success of 45S5 bioactive glass as the first member of the bioactive glass family (45 wt% SiO2, 24.5 wt% CaO, 24.5 wt% Na2O, and 6.0 wt% P2O5), bioactive glasses with different chemical formulations were introduced to the biomaterial market as remineralizing agents [5]. The mechanism of action of bioactive glasses in vivo or in simulated body fluids can be summarized as a superficial reaction in some phases of release and exchange of ions, dissolution of glass network, and deposition of calcium and phosphorous in the form of a hydroxyapatite layer [6].

In the recent years, biomedical polyurethane elastomers with improved hydrolytic stability were synthesized. Due to improved biocompatibility, durability, toughness, and biostability, they are considered as part of biomedical implants [7]. Polyurethanes are often synthesized by a reaction between isocyanate and polyol molecules in the presence of a catalyst [8]. Canali et al. [9] used a desensitizing varnish containing polyurethane polymer. They showed the better adhesion of polyurethane to tooth compared with common synthetic resin used in fluoride varnishes. The polymer material used in this study was urethane polypropylene glycol oligomer (UPGO) that contains polypropylene glycol 400, which is a polymer of repeating units of polypropylene oxide, and is used as a polyol in combination with isophorone diisocyanate (IPDI) for the synthesis of polyurethane. Propylene glycol is a humectant commonly used in the composition of toothpastes [10]. It is a viscous alcoholic compound commonly used as a vehicle for the delivery of calcium hydroxide and some sealers into the root canal system and also to improve the handling properties of mineral trioxide aggregate. It is less toxic compared with other vehicles and has been approved by the American Food and Drug Administration. Its penetration into dentin is higher than distilled water. Also, it has optimal antibacterial activity [11, 12].

Bioactive glasses are high soluble materials [6]. Phosphoric acid or deionized water has been used in combination with bioactive glass powder to obtain a paste-like consistency for easier application on the tooth surface [13]. However, poor manipulation is still one of the disadvantages of these materials in dental applications. Thus, in order to obtain suitable clinical handling, enable sustained ion release, prevent the possible wash out of bioactive glass, and preserve its optimal properties at the same time, we used UPGO in combination with bioactive glass in this study.

This study aimed to assess the efficacy of a composite paste (prepared by mixing a polymer liquid with bioactive glass powder for easier application) for remineralization of demineralized enamel.

## 2. Materials and Methods

### 2.1. Preparation of Bioactive Glass Paste

#### 2.1.1. Bioactive Glass Powder Synthesis

Bioactive glass (52S4) with a formulation presented in Table 1 was prepared by the melt-quenching technique. In this technique, the primary requirements to provide bioactive glass oxides included CaCO3, Na2CO3, SiO2, and P2O5 powders (Sigma-Aldrich, Gillingham, UK). The primary ingredients in the required amounts were added to a ball mill in acetone environment and mixed at 200 rpm for 2 h. They were then dried at 70°C for 2 h. The obtained powder was melted in an alumina furnace at 1400°C for 2 h followed by carbonate elimination at 950°C for 5 h. The melted material was placed on a metal surface to cool down and yield amorphous glass. The obtained frits were ground in a ball mill at 250 rpm for 3 h to obtain glass powder. The mean particle size was measured to be 5 *μ*m using Analysette 22 Comfort laser particle sizer (FRITSCH, Germany). Also, 90% of the particles were smaller than 12 *μ*m.

### 2.2. Liquid Synthesis

For liquid synthesis, 0.12 mol polypropylene glycol (equal to 48 g) along with 0.1 mol isophorone diisocyanate (equal to 22.2 g) was heated in a reactor equipped with a mechanical stirrer at 80°C for 2 h in the presence of 0.01 g stannous octoate. To prevent oxidation and color change of urethane polymer, nitrogen atmosphere was used. For purification, the obtained polymer was dissolved in 100 mL chloroform and deposited by using 200 mL diethyl ether solvent. The deposits were dried in a vacuum oven at 40°C for 2 h. The obtained urethane polymer was 65 g, and the reaction efficiency was around 93%. The obtained polymer powder was mixed with ethanol solvent to reach 50% solid percentage. The obtained pure polymer solution was used for other tests.

Gel permeation chromatography analysis was used to evaluate the number average molecular weight (Mn) and weight average molecular weight (Mw) molecular weights of the synthesized polymer. The reaction proceeded until the desired molecular weight of the polymer was obtained. Chemical structure and functional groups were analyzed and confirmed by Fourier-transform infrared spectroscopy (FTIR) and nuclear magnetic resonance (NMR).

**Figure 1:**
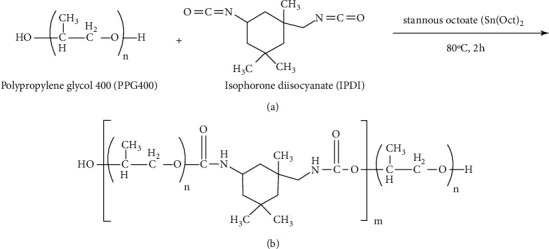
Synthesis reaction (a) and structure of UPGO structure (b).

The obtained NMR spectra were recorded using Bruker DRX-500 AVANCE (500.1 MHz for 1 H and 125.0 MHz for 13°C). The spectra were measured in DMSO-d6 as a solvent. FTIR (PerkinElmer, Model Frontier®, USA) was conducted on the pellets that were prepared by mixing the proper amounts of the powder with KBr. The transmittance spectra were reported with a wavenumber resolution of 4 cm^−1^ in the range of 400–4000 cm^−1^.  Figure 1 presents the composition of the primary ingredients and the obtained urethane polymer.

### 2.3. Sample Preparation

Sound extracted human third molars were used in this study (45 samples for the microhardness test, and the remaining for characterization tests). The teeth had no caries, cracks, or anomalies. Also, the selected teeth had almost similar dimensions. The teeth were immersed in 0.5% chloramine T solution at 4°C for 7 days. Next, they were cleaned with rubber cup and pumice paste using low-speed handpiece. The teeth were visually inspected in terms of caries, enamel cracks, and cervical and occlusal wear under unit light. Teeth with such defects were excluded and replaced. The roots were then cut at the cementoenamel junction by a diamond disk under air-water coolant. The crowns were mounted in polyester. Enamel segments with an approximate thickness of 1.5 mm were cut out of the buccal surface of the mounted tooth crowns by a cutting machine (Mecatome T201A; Presi, France). The enamel surfaces were polished with 320-, 600-, and 1200-grit abrasive papers (Matador, Germany) to obtain a smooth surface.

### 2.4. Demineralizing and Remineralizing Processes

The demineralizing solution contained 2.2 mM CaCl2, 2.2 mM NaH2PO4, and 0.05 M acetic acid. pH was adjusted to 4.4 using 1 M KOH [14]. To determine the appropriate time required for demineralization, a pilot study was carried out on three enamel samples. The samples were stored in the demineralizing solution for 3, 7, 10, and 14 days. After assessing the degree of demineralization of the samples by the microhardness test, 14 days were found to be the appropriate period for demineralization, which was in agreement with the methodology of Milly et al. [15]. Accordingly, sound samples were immersed in the demineralizing solution for 14 days. To simulate the oral environment temperature, the samples were incubated at 37°C (Memmert GmbH Co. KG., Germany). The demineralizing solution was refreshed daily.

The remineralizing solution (artificial saliva) contained 1.5 mM CaCl2, 0.9 mM NaH2PO4, and 0.15 M KCl at a pH of 7 [14]. The demineralized samples were immersed in the remineralizing solution for 14 days and incubated at 37°C during this time period. The remineralizing solution was refreshed daily.

### 2.5. Vickers Microhardness Test

For the Vickers microhardness test, the smooth enamel surfaces were coated with acid-resistant varnish (nail varnish) except for a window measuring 3 × 2 mm. This was done to confine the testing area. The primary microhardness of all samples was measured by creating three indentations on each surface by applying 100 g load for 8 s by the Vickers microhardness tester (BARESIS D-89610V Test, Germany). The mean of the three values was calculated and reported as the Vickers hardness number (VHN) of each sample. The samples underwent demineralization process as mentioned above and were analyzed again. Then, the enamel surface treatments were done as follows:

- FluoroDose® group: fluoride varnish (FluoroDose®, Centrix Inc., USA) was applied on demineralized enamel samples according to the manufacturer's instructions. One layer of self-etch bonding agent (G-Bond; GC, Tokyo, Japan) was applied on the samples and light-cured for 20 s by a curing unit with a light intensity of 1200 mW/cm2.
- GC Tooth Mousse group: The CPP-ACP paste (GC Tooth Mousse; Recaldent, GC Corporation, Tokyo, Japan) was applied on the surface of the samples according to the manufacturer's instructions. The bonding agent was applied and cured.
- 52S4/UPGO group: 52S4 bioglass powder and UPGO synthetic polymer were mixed in a weight ratio of 2 : 1, respectively, to obtain a paste with creamy consistency. A sufficient amount of this paste was applied on the surface of the samples. Then, bonding agent was applied and cured.  Table 2 shows the instruction for use and composition of remineralizing agents.

As mentioned above, the samples were immersed in remineralizing agent for 14 days and were analyzed again.

### 2.6. Enamel Characterization

Similar to the microhardness test, the samples were analyzed in three steps (sound, demineralized, and remineralized). XRD (X′Pert PRO MPD, PANalytical, Netherlands) was performed to analyze the crystalline structure of the material. The diﬀraction pattern was determined using a diﬀractometer with a copper anode (40 kV, 40 mA, and step size of 0.02 in the range of 5–120° 2Ѳ). Field emission scanning electron microscopy (FE-SEM; Mira3Tescan-MU, Czech Republic) coupled with EDS (SAMX, France) with an accelerated voltage of 15 kV was performed to evaluate the enamel morphology and compositional changes. For image clarity, the samples were sputter-coated with gold. EDS was performed in three points such that three points in each sample were analyzed, and the mean percentage of the three values was calculated and reported.

### 2.7. Statistical Analysis

The microhardness test results were analyzed by repeated measures ANOVA followed by one-way ANOVA and Tukey's post hoc test (*P* < 0.05).

## 3. Results

### 3.1. Bioactive Glass Characterization

**Figure 2:**
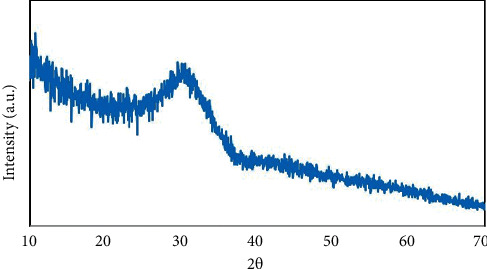
XRD pattern of 52S4 bioactive glass.

Figure 2 shows the XRD pattern of 52S4. The broad hump at 20–30° 2ϴ is characteristic for an amorphous glass [16].

### 3.2. UPGO Characterization

#### 3.2.1. GPC

According to the GPC results, UPGO with Mn of 7600 Da, Mw of 12920 Da, and a polydispersity index (PI) of 1.7 was synthesized.

#### 3.2.2. FTIR

**Figure 3:**
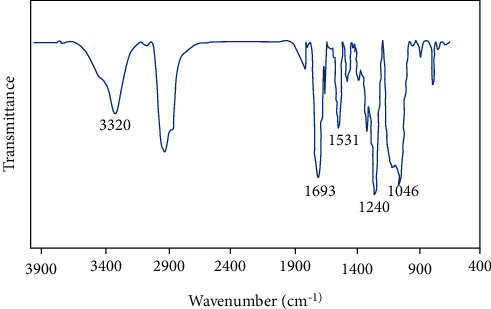
FTIR spectrum of UPGO.

Figure 3 shows the FTIR spectrum of UPGO. The peak at 3320 cm^−1^ is related to N-H stretching and indicates the formation of urethane linkages. The C-H stretching vibration was located at around 2800–3000 cm^−1^. The peak at 1693 cm^−1^ was ascribed to the C = O stretching absorption in polyurethane group. The 1531 cm^−1^ peak was related to the N-H bending vibration in polyurethane group. Moreover, the polymerization of urethane bond was indicated by the disappearance of the peak related to isocyanate group. The absence of free NCO group at 2250–2270 cm^−1^ confirmed the complete reaction of diisocyanate with polyol, which led to the formation of polyurethane. The presence of C-O bonds in structure was indicated by 1240 and 1046 cm^−1^ peaks.

#### 3.2.3. NMR

**Figure 4:**
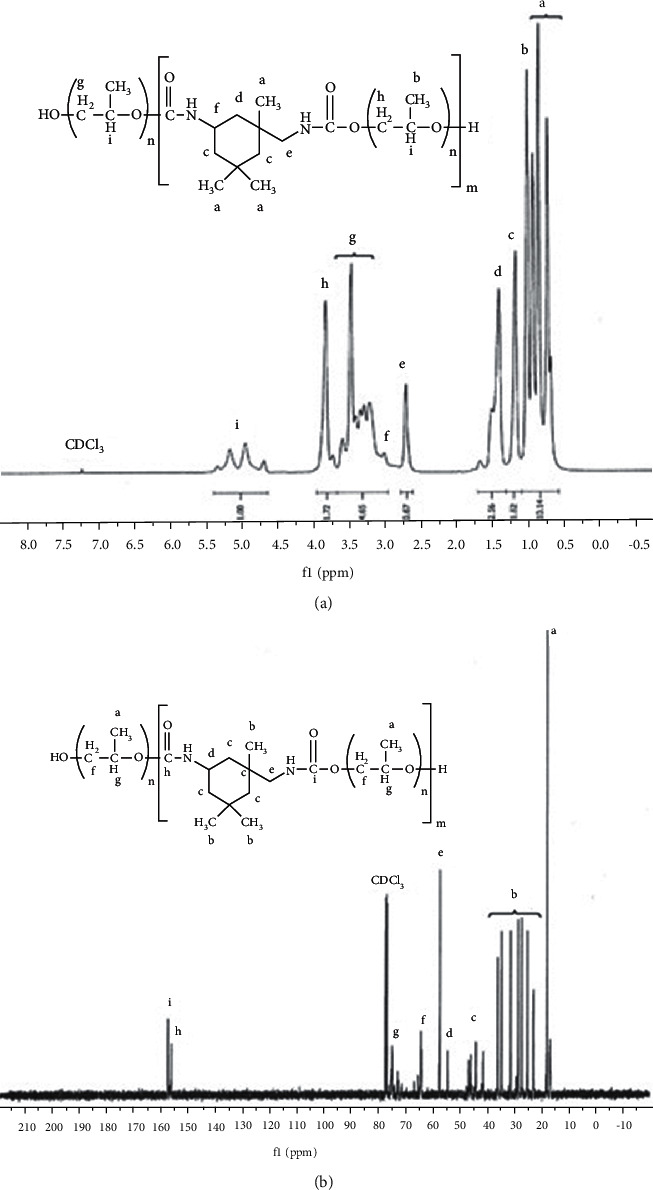
^1^H NMR (a) and ^13^C NMR spectra (b) of UPGO.

The chemical composition of UPGO was recognized by ^13^C and ^1^H NMR spectral data (Figure 4). Carbons in methyl groups of polypropylene glycol (PPG) appeared at *δ* 18 ppm, while those related to methyl branches of IPDI ring appeared as multiple peaks at *δ* 22–35 ppm. The multiple peaks located at *δ* 42–47 ppm were assigned to CH_2_ carbons in IPDI ring. The ^13^C-NMR spectrum of UPGO showed signals at *δ* 54 ppm and *δ* 57 ppm, which were related to carbons of the CH group in IPDI ring and carbon of the methylene group in IPDI connected to the urethane group. The CH_2_ and CH group carbons related to the repeating units of PPG were observed at *δ* 64 ppm and *δ* 74–76 ppm, respectively. The carbons related to carbonyl groups in urethane groups appeared as two close peaks at *δ* 156 ppm and *δ* 157 ppm. The protons related to the methyl groups in the IPDI ring and methyl branches in repeating units of PPG appeared at *δ* 0.6–0.8 ppm and 1.2 ppm, respectively. The peaks at *δ* 1.3 and 1.5 ppm can be ascribed to the protons of the CH_2_ groups in the IPDI ring. The methylene group protons in the IPDI ring linked to the urethane group appeared at *δ* 2.7 ppm. Furthermore, the CH protons in the IPDI ring appeared as a weak peak at *δ* 3.1 ppm. Multiple wide peaks around *δ* 3.4–3.6 ppm were related to the methylene group protons of repeating units of PPG, which had no linkage to the urethane group. The methylene group protons related to the PPG units linked to the urethane group were located at *δ* 3.8 ppm and indicated the linkage between the PPG oligomers by the IPDI. The ^1^HNMR signals split to several peaks at *δ* 5 ppm were assigned to the CH group protons of the PPG repeating units [17].

### 3.3. Microhardness Test

According to the results of the Vickers microhardness test presented in Table 3, the microhardness of sound samples was not significantly different in the three groups (*P* > 0.05). However, within each group, sound, demineralized, and remineralized samples had a significant difference with each other in microhardness. Also, samples remineralized with bioactive glass had significantly higher microhardness than the fluoride (*P* < 0.05) and CPP-ACP (*P* < 0.05) groups. However, the difference in this respect was not significant between the CPP-ACP and fluoride groups (*P* > 0.05).

### 3.4. Enamel Characterization

#### 3.4.1. XRD

**Figure 5:**
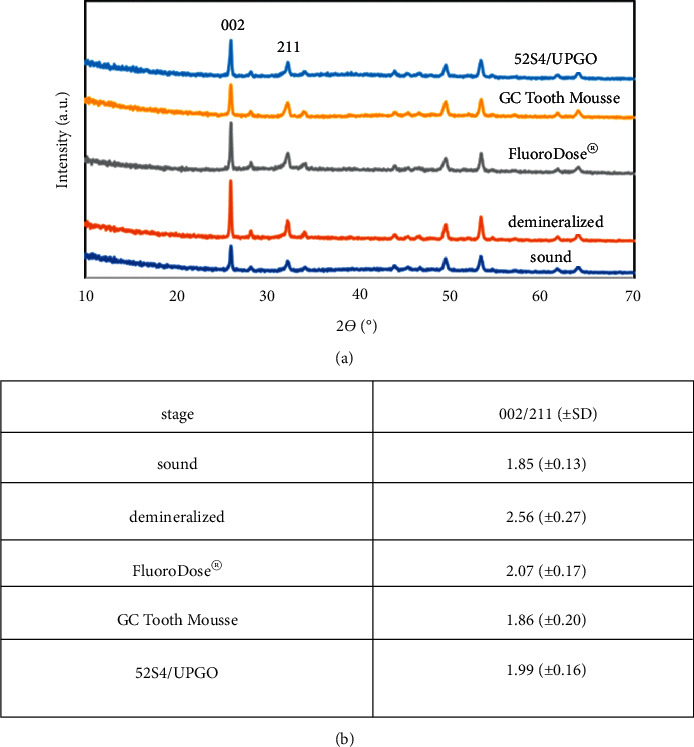
XRD patterns (a) and 002/211 ratios of enamel specimens at different stages (sound, demineralized, and after remineralization by FluoroDose®, GC Tooth Mousse and 52S4/UPGO, *n* = 5, SD = standard deviation).

Considering the uniqueness of each enamel sample in XRD analysis, each group of remineralized samples was compared with the XRD results of the same sound and demineralized enamel samples (Figure 5). The 002 peaks at 2*θ* = 25.8 and 211 peak at 2*θ* = 31.8 were exclusive to hydroxyapatite according to the basic reference information of XRD patterns. To better compare the patterns, the ratio of the intensity of two main peaks (002/211) was calculated. The closer the ratio of remineralized and sound samples, the higher the efficacy of the respective material for remineralization of demineralized sample would be.

#### 3.4.2. FE-SEM/EDS

**Figure 6:**
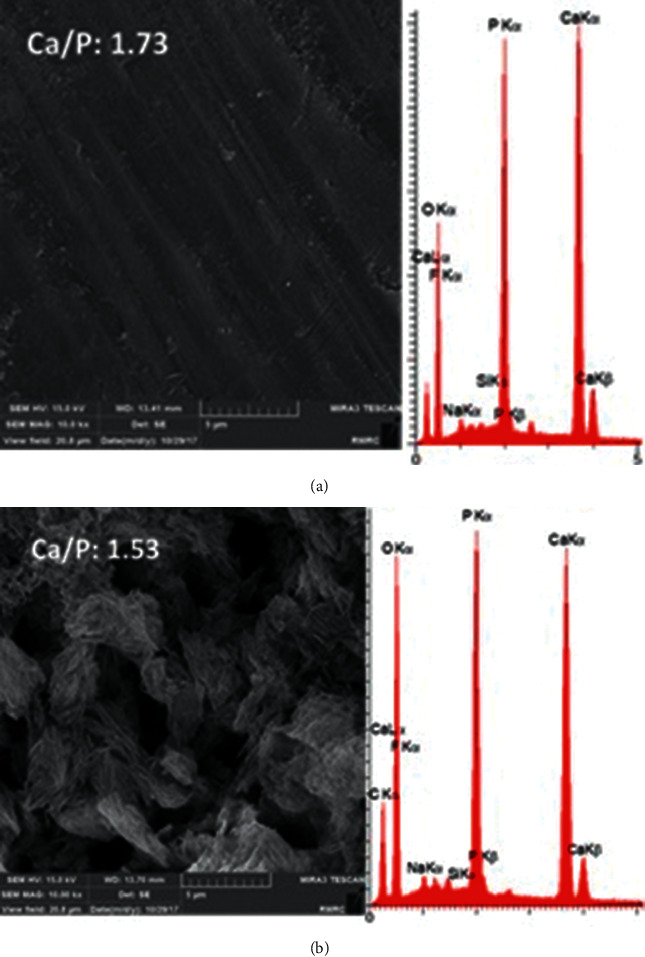
FESEM/EDS analyses of sound and demineralized enamels.

**Figure 7:**
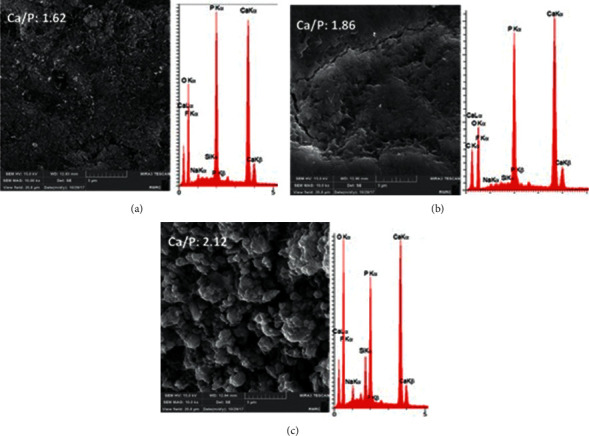
FESEM/EDS analyses of remineralized enamels by FluoroDose® (a), GC Tooth Mousse (b), and 52S4/UPGO (c).

In FE-SEM, the samples were observed at x10000 magnification (Figures 6 and 7). The EDS analysis was performed at three points, and the mean value was calculated. The atomic percentage ratio of calcium to phosphorus was calculated (Figures 6 and 7). According to the FE-SEM images, the sound enamel had a smooth surface with no porosities. FE-SEM images of demineralized enamel well illustrated the enamel surface porosities caused by the acid attack. Images of the samples remineralized with fluoride showed remineralization of demineralized enamel characterized by filling of porosities by the formed fluorapatite.  Figure 7(b) shows enamel samples remineralized with CPP-ACP. Remineralization occurred by the formation of new hydroxyapatite crystals due to the presence of calcium and phosphorous ions. But, a uniform complete deposition was not seen in any image.  Figure 7(c) illustrates an enamel sample remineralized with bioglass paste and deposition of hydroxyapatite with higher density. The calcium/phosphorous ratio of each image was identified. GC Tooth Mousse and 52S4/UPGO showed higher ratio compared with FluoroDose®.

## 4. Discussion

High solubility of glass, which is the main factor responsible for its bioactivity and biomineralization properties, shortens the durability of the material on the tooth surface and increases the possibility of its washout [18]. In this study, UPGO synthetic polymer was used in combination with bioactive glass powder to obtain an optimal consistency and preserve the properties of the material. This polymer can remain on the tooth surface and harden. Thus, its contact with the tooth structure is prolonged. The reason for application and curing of a bonding layer on the remineralizing agent is to standardize the condition of teeth in terms of their contact with the remineralizing agents with different solubility rates in the saliva. The application of self-etch adhesive containing hydrophilic HEMA does not limit the diffusion of water and soluble remineralizing ions [19]. The important topic when comparing the three materials is to promote the remineralization of demineralized enamel. Microhardness test is a reliable method for examining the mineral content of enamel following remineralization and is used in dental studies [20]. According to the obtained results, the increase in microhardness caused by bioactive glass was significantly greater than that caused by fluoride varnish and CPP-ACP. However, CPP-ACP and fluoride had no significant difference in this respect. According to our results and those of previous studies, in demineralized groups, the 002 peak related to the crystal plate of hydroxyapatite had a higher intensity than the other three groups [21, 22]. The 002 hydroxyapatite peak is intensified when the degree of C-axis orientation of hydroxyapatite crystals increases. Hydroxyapatite crystals in demineralized enamel are removed faster from the peripheral prisms than the central prisms. Thus, hydroxyapatite crystals at the center are organized along the C axis and increase the intensity of 002 peak related to the crystal plate of hydroxyapatite of demineralized enamel [22, 23]. The results of XRD were in agreement with FESEM images. Images of demineralized enamel indicated porous structure of hydroxyapatite crystals. SEM studies on demineralization process have shown that demineralization initiates from the interface of the center of prism or rod with the prism sheath, and progresses along the C axis in an anisotropic manner [24]. The results of EDS revealed higher calcium to phosphorous ratio in bioactive glass, which was in agreement with the microhardness test results.

The role of fluoride (bioactive component of FluoroDose varnish) in conferring resistance to the enamel against demineralization is mediated by the replacement of OH^−^ in hydroxyapatite with fluoride ion and subsequent formation of acid-resistant fluorapatite [25]. The fluoride is absorbed by partially demineralized crystals, leading to calcium uptake by the surface of these crystals [26]. Nonetheless, fluoride cannot penetrate deep into the subsurface layers of demineralized enamel and can only cause superficial enamel remineralization [27]. Bakry et al. [28] measured the microhardness of enamel remineralized with bioglass and fluoride. They reported significantly higher microhardness of enamel samples remineralized with bioactive glass compared with fluoride in subsurfaces. Furthermore, the effect of fluoride is dose-dependent. In fact, the formation of fluorapatite is a self-limiting phenomenon, which prevents the penetration of calcium and phosphate into deeper layers [29]. Evidence shows that CPP-ACP (bioactive component of GC Tooth Mousse) causes greater remineralization than fluoride, which has been attributed to the availability of calcium and phosphate ions [30, 31]. CPP stabilizes the calcium phosphate on the enamel surface such that in a metastable solution, it attaches to calcium phosphate and prevents its dissolution. CPP-ACP serves as a source of calcium phosphate and maintains the calcium and phosphate ions in a supersaturated state [29, 31]. On the other hand, evidence shows that remineralizing agents with small particle size have higher capability in penetration into the porosities of demineralized enamel [32]. The size of Recaldent CPP-ACP or CPP-ACPF clusters is about 2 nm, which is smaller than the size of bioactive glass particles used in this study. In general, it may be stated that in contrast to fluoride, CPP-ACP remineralizes both the superficial and subsurface layers [33]. In comparison with CPP-ACP and bioactive glass (52S4, the bioactive component of experimental material), lower microhardness of CPP-ACP group can be due to the amorphous nature of this material and its inability to bond to tooth structure [34]. When bioactive glass is exposed to the saliva, ion exchange occurs between the sodium and hydrogen ions. The local pH rises, and a silica-rich layer forms on the surface of bioactive glass. Migration of calcium and phosphate ions in the bioactive glass and also saliva through this layer to the surface leads to the formation of amorphous calcium phosphate. Eventually, crystallization and conversion to hydroxyapatite occur [35]. Silica-rich can serve as a nucleation site for reuptake of calcium and phosphate ions [34]. It appears that adhesion of UPGO polymer can enhance enamel remineralization effect of bioactive glass without interfering with its mechanism of action.

## 5. Conclusion

All three remineralizing agents increased the microhardness of demineralized enamel, but this increase was greater by bioactive glass compared with fluoride and CPP-ACP. However, the results of fluoride and CPP-ACP groups were not significantly different. Remineralization efficiency of 52S4-UPGO was also confirmed by FESEM micrographs, which showed more dense hydroxyapatite formation and also with a XRD pattern closer to sound enamel. We obtained a paste with suitable consistency by combining bioactive glass with UPGO, which had easy application on the tooth surface and preserved its remineralizing potential as well.

## Figures and Tables

**Table 1:** Bioactive glass composition (mol%).

SiO2	Na2O	CaO	P2O5

52.3	13.7	32.3	1.7

**Table 2:** Instruction for use and composition of remineralizing agents.

Material	Manufacturer	Ingredients	Instruction for use

52S4/UPGO	Experimental	Bioactive glass/synthetic polymer	Mixing ratio: 2 : 1 (powder:liquid); apply on the enamel surface for 5 min
GC tooth mousse	GC corp., Tokyo, Japan	Water, glycerol, CPP-ACP (10%), D-sorbitol, xylitol, sodium carboxymethyl cellulose, propylene glycol, silica, titanium dioxide, zinc oxide, phosphoric acid, magnesium oxide, sodium saccharin, ethyl p-hydroxybenzoate, butyl p-hydroxybenzoate, propyl p-hydroxybenzoate, and guar gum	Apply a sufficient paste on the enamel surface for 3 min.
FluoroDose®	Centrix Inc., USA	5% NaF, rosin, xylitol	Apply a thin film of varnish on the enamel surface and let the varnish dry for approximately 10 seconds.
G-BOND^TM^	GC Corp., Tokyo, Japan	Phosphoric acid monomer, 4-MET, UDMA, TEGDMA, photo-initiator, stabilizer, fumed silica filler, acetone, water	Apply to the enamel surface and leave undisturbed for 5–10 seconds after the end of application. Then, dry thoroughly for 5 seconds under maximum air pressure and light cure for 10 s.

CPP-ACP: casein phosphopeptide-amorphous calcium phosphate; 4-MET: 4-methacryloxyethyl trimellitic acid; TEGDMA: triethylene glycol dimethacrylate; Bis-GMA: bisphenol A-glycidyl methacrylate; UDMA: urethane dimethacrylate.

**Table 3:** Means and standard deviation of microhardness values for each group (at sound, demineralized, and remineralized stages).

Groups	Stages	N	Mean	Std. deviation

52S4/UPGO	Sound^a^	15	343.33	12.82
	Demineralized^b^	15	97.20	7.76
	Remineralized^c^	15	224.20	12.43

FluoroDose®	Sound^a^	15	355.20	37.11
	Demineralized^b^	15	113.80	35.49
	Remineralized^d^	15	174.33	35.78

GC tooth mousse	Sound^a^	15	343.00	33.51
	Demineralized^b^	15	115.73	40.77
	Remineralized^d^	15	182.53	37.34

^*∗*^Different lower-case letters show statistical difference.

## Data Availability

The data used to support the findings of this study are included within the article.
